# ABA and Not Chilling Reduces Heat Requirement to Force Cherry Blossom after Endodormancy Release

**DOI:** 10.3390/plants11152044

**Published:** 2022-08-04

**Authors:** Frank-M. Chmielewski, Klaus-Peter Götz

**Affiliations:** Agricultural Climatology, Faculty of Life Sciences, Humboldt-University of Berlin, Albrecht-Thaer-Weg 5, 14195 Berlin, Germany

**Keywords:** *Prunus avium* L., cv. Summit, sweet cherry, phenology models, ecodormancy, chill, forcing requirement, abscisic acid

## Abstract

Models used to predict the onset of fruit tree blossom under changed climate conditions should be physiologically based as much as possible. Pure optimized phenology models carry the risk of unrealistic predictions due to a misinterpretation of metabolic processes. This was the motivation determining the relevant phases for chill and heat accumulation, which induces cherry blossom (cv. Summit). Investigations are based on 8 years of observational and analytical data, as well as on controlled experiments. For ‘Summit’ buds, to be released from endodormancy, 43 chill portions from 1 September are necessary. After endodormancy release (t_1_), on average on 30 November, no further chilling is required, because no correlation between chill accumulation during ecodormancy and the subsequent heat accumulation until ‘Summit’ blossom exist. The declining amount of heat, which induces cherry blossom after t_1_—shown in several forcing experiments—seems to be the result of the declining bud’s abscisic acid (ABA) content, up to ~50% until the beginning of ontogenetic development. Shortly after t_1_, when the bud’s ABA content is high, a huge amount of heat is necessary to induce cherry blossom under controlled conditions. Heat requirement reduces during ecodormancy along with the reduction in the ABA content. According to these findings, plant development during ecodormancy is suppressed by low temperatures in the orchard and a slowly declining bud’s ABA content. These results should lead to a better consideration of the ecodormancy phase in phenology models.

## 1. Introduction

Temporal cessation of flower bud’s growth in autumn is a strategy of perennials to acquire cold and freeze tolerance, as well as to protect the meristematic cells of the developing organs against winter temperatures. This phase is defined as dormancy, which is characterized by reduced metabolic activity [1,2] due to decreased plasmodesmal connectivity [3] and no recognizable structural development [4]. Lang et al. [5] divided dormancy into para-, endo- and ecodormancy. In autumn, the transition from paradormancy (summer dormancy) to endodormancy begins with the cessation of bud growth under the influence of declining temperatures and/or daylengths [6]. For Prunus sp. the effect of photoperiod is probably rather insignificant [7]. In temperate climates, ‘leaf fall’ could be a good indicator for the start of endodormancy phase [8,9], the true winter rest in which growth and development of buds is impossible, even under favorable environmental conditions. During the endodormancy phase, buds accumulate a certain amount of chill (individual chill requirement), necessary to break it. When endodormancy is released, bud growth potential is resumed, but growth and development remain still suppressed due to hostile weather conditions in winter. This phase is defined as ecodormancy (quiescence), in which the energy metabolism in the form of glycolysis and tricarboxylic acid cycle is shut down to minimum [10]. It keeps the reproductive organs biologically silent until spring when the water content in the buds increases, due to continuously rising air temperatures [8]. The final phase until bud burst or flowering is the ontogenetic development, which is characterized by the upregulation of the carbohydrate and energy metabolism. It is important to recognize that ontogenetic development must start a few weeks before bud swelling, because it marks the irreversible start of bud development [11]. These phenological phases should be adequately considered in phenology models.

The models can be divided into two main categories:

(1) Pure forcing or 1-phase models, e.g., thermal time or spring warming models ([12] and the references therein), only consider the heat accumulation during the ontogenetic phase in spring. The advantage of these models is that they do not have to take the dormancy phases into account. However, they should only be used if endodormancy is assuredly released before heat accumulation starts. A challenge of these models is the precise definition of the starting date of forcing accumulation, according to our notation referred as t_1_*, which cannot be observed on the tree and is thus mostly an optimized model parameter and not physiologically based.
(2) Chilling/forcing or 2-phase models, which can be realized as sequential or parallel models, try to consider both the dormancy and growth phase in order to calculate the timing of phenological events ([12] and the references therein). This approach is justified, because air temperature and/or photoperiod are considered to be the driving factors for the induction, maintenance and release of dormancy. Shortening daylength and/or low temperature signals are responsible for initiation and development of endodormancy in autumn [13]. However, after the induction of endodormancy, air temperature is the most important factor to leave this state in favor of resumption of growth potential [9]. Thus, phenological models assume that a sufficient amount of chill temperatures are needed for endodormancy release, which is highly variable between tree species, provenances and cultivars [14,15]. Afterwards, by elevated air temperatures in spring, the subsequent quiescent phase of ecodormancy is overcome and results in the beginning of ontogenetic development. However, even for this category of models, determining the exact date of endodormancy release (t_1_) and the beginning of ontogenetic development (t_1_*) is challenging, because these parameters are predominantly statistically derived. Sequential phenology models assume that forcing temperatures are only effective if a certain chill requirement of the plant is met, i.e., endodormancy is released [16]. In parallel models [17,18,19,20], chill and heat are accumulated simultaneously with the assumption that a lack of chilling can be substituted by a higher forcing amount and vice versa (Equation (1)). In these models, chilling is usually calculated from autumn of the previous year (September or November) and forcing from January or February, both until the onset date of the phenological spring event [17,21,22,23].

(1) $$F*=\;a+b\;\cdot \exp\;\left(c\;S_{c}(t)\right),\;\mathrm{where}\;a,b>0\;\mathrm{and}\;c<0$$

F* is the forcing requirement, depending on the state of chilling *S_c_*(*t*) at the time *t*.

The negative exponential relationship between chill and heat accumulation (Equation (1)) is confirmed in several experiments under controlled conditions, until present [24,25,26], but beyond that, further physiologically based experiments are indispensable [11,23,27]. An intermediate model between the sequential and parallel approach is the chill overlap model [20,28,29], which assumes that for a certain time chill accumulation is still relevant after t_1_. For three *Prunus dulcis* cultivars (almond) in California, a 75% chill overlap was found. This means that chill accumulation continued until ~75% of the heat requirement was met, necessary to force bloom [28]. For parallel and even sequential phenology models, the risk to overestimate the bud’s chill requirement is high, if t_1_ is unknown or not exactly determined. This would have strong consequences if these models were used later to estimate the effect of global warming on phenological events [11,30,31,32]. 

Meanwhile, it is known that in deciduous trees the phytohormone abscisic acid (ABA) has a growth suppressing function. In particular, in the last few years physiological and transcriptomic studies have proposed the central role for ABA in the metabolic inhibition of bud ‘activity’ during winter rest [8,33,34]. A number of different genes are involved in the biosynthesis of ABA [35]. For aspen, ABA seems to have an important function in the establishment of endodormancy, since the blocking of cell to cell communication through plasmodesmata is mediated by ABA [36]. Thus, the transport of growth-promoting signals during endodormancy is suppressed. To escape this state, buds need to be exposed to colder temperatures. Vimont et al. [34] reported for sweet cherry that endogenous ABA at the date of ‘dormancy release’ show a good match for two cherry cultivars, ‘Cristobalina’ and ‘Regina’, respectively. The ABA levels were low before dormancy onset but as they increase dormancy is triggered. High ABA levels maintain dormancy and endodormancy is released as ABA content declines. Similar results were found for peach and pear [13,37]. Thus, emerging metabolites and intermediates and their resulting ‘signaling effects’ can have a controlling influence on the transitions of phenological phases.

In this paper, we present the timing and duration of the ecodormancy phase for the sweet cherry cultivar Summit in 8 years. The timing of this phase was derived from analytic work, supported by experiments under controlled conditions and metabolomic studies. The hypothesis of this study is that during ecodormancy the declining ABA content in cherry flower buds and not chilling reduces the heat requirement until blossom. It is shown that a ~50% reduction in the ABA content is related to the beginning of ontogenetic development. The negative exponential relationship between chill and heat accumulation in parallel phenology models seems to be an approximation for the declining ABA content in the buds, which is usually not analyzed and thus not yet considered in phenology models. This study is intended to stimulate a physiological revision of phenological modeling approaches.

## 2. Results

### 2.1. Average Timing and Duration of Dormancy Phases and Ontogenetic Development for ‘Summit’ Flower Buds

After ‘total leaf fall’, on average on 6 November (310 DOY), photosynthesis and the assimilation of the tree come to a standstill, so that the tree entered into the endodormancy phase (Table 1). A cultivar specific chilling requirement must be fulfilled to leave this state. Our climate chamber experiments showed that for ‘Summit’, endodormancy release (t_1_) takes place relatively uniformly at the end of November or beginning of December, on average on 30 November (DOY 334 ± 6.8 d). Thus, the endodormancy phase for ‘Summit’ (LF–t_1_) lasted at our site ~25 ± 5.1 days. Starting on 1 September (244 DOY), a relatively constant number of chill units was accumulated until t_1_ (42.6 ± 3.3 CP on average, Table 2). The duration of the subsequent ecodormancy phase (t_1_–t_1_*) is ~83 ± 17.8 days. The higher temporal variability of this phase mainly resulted from the variable start of ontogenetic development (t_1_*: DOY 52 ± 15.9 d, CV = 30.6%), depending on the course of air temperature, mainly in February or March. This indicates that a fixed date for the start of heat accumulation (e.g., 1 January or 1 February) is a strong simplifying assumption in phenology models. Finally, the beginning of blossom was observed on 15 April (105 DOY). Since we defined t_1_* as the date when the bud’s water content started to rise continuously, the phase of ontogenetic development (t_1_*–BB) lasted 53 ± 11.3 days. The significant positive correlation between the timing of t_1_* and BB (r = 0.76, *p* ≤ 0.05) supports the relatively constant forcing accumulation FA(t_1_*–BB) during ontogenetic development (Table 2).

### 2.2. Average Chill and Heat Accumulation of ‘Summit’ Flower Buds during Dormancy Phases and Ontogenetic Development

To leave the endodormancy phase, buds need some cold stimulus, which can be calculated in chill portions. According to the definition of dormancy phases [5], chilling should be relevant until t_1_, the date of endodormancy release. From this point on, buds are able to accumulate heat, which finally forces the beginning of blossom. This has been clearly demonstrated in the climate chamber experiments during 8 seasons, described in Section 4.3. 

It is common, that the starting date for chill accumulation in temperate climates is 1 September (244 DOY), because air temperatures in August are still too high to contribute to chilling. Since parallel phenology models assume that chilling and forcing can compensate for each other, Table 2 also lists the accumulated chill units (in CP) during ecodormancy (t_1_–t_1_*) and ontogenetic development (t_1_*–BB). The accumulation of forcing units (in GDH) was calculated for the ecodormancy phase and ontogenetic development; in the latter, forcing must be relevant.

As already mentioned, the average chilling requirement of ‘Summit’ is ~43 ± 3.3 CP (range 40–49 CP). However, during ecodormancy another 50 ± 10.1 CP were available, which theoretically could be accumulated by the buds (Table 2). Even in the phase of ontogenetic development (t_1_*–BB), further 37.5 ± 10.7 CP were calculated. However, this chill amount cannot be developmental orientated, since during this phase the water content in the buds already rises, indicating biological activity and proceeding bud formation. Thus, between 1 September and beginning of cherry blossom on average 130.1 ± 8.0 CP are theoretically available. 

According to the annual course of air temperature, between 312 GDH (2016/17) and 2460 GDH (2015/16) were accumulated during ecodormancy (mean ~885 ± 654.4 GDH). The coefficient of variation (CV = 74%) points to a high interannual variability during this phase (see also Figure S1). In contrast, the accumulated GHDs between t_1_* and BB were relatively constant, averaging ~3447 ± 357 GDH. CV decreased from 74.0% (t_1_–t_1_*) markedly to 10.4% (t_1_*–BB). If we postulate that the heat requirement to force cherry blossom should be nearly constant for a specific cultivar and location, t_1_* must be the right starting date for heat accumulation. The high forcing amount of 2460 GDH during ecodormancy in the 2015/16 season (Table 2) did not accelerate the beginning of cherry blossom (111 DOY, Table 1). Conversely, the low forcing amount in 2016/17 of 312 GDH did not result in a very late onset of blossom, rather in an early one (97 DOY, Table 1). Thus, if one assumes that forcing between t_1_* and BB can compensate for a deficit of chilling during ecodormancy (t_1_–t_1_*), a negative correlation between both parameters should exist. In our study, no correlation between both phases were found (r = 0.09^ns^, Table 2). Likewise, no significant correlation was found between CA (244–BB) and FA (t_1_*–BB), r = −0.11^ns^. The significant correlation coefficient between chill and heat accumulation during ecodormancy (r = 0.76*, *p* ≤ 0.05) only indicates that, in this phase, air temperature contributes for both chilling and forcing. This emphasizes how important it is to know the exact timing of dormancy phases in order to avoid misinterpretations.

### 2.3. Substitution of Chilling and Forcing under Controlled Conditions

As described in Section 4.4, in the 2018/19 season, a climate chamber experiment was performed in order to investigate the compensatory effect between chill and heat accumulation of ‘Summit’ buds. The experiment started at the date of endodormancy release on 29 November 2018 (333 DOY, Table 1), when buds received 40 CP in the orchard (Table 2).

**Figure 1:**
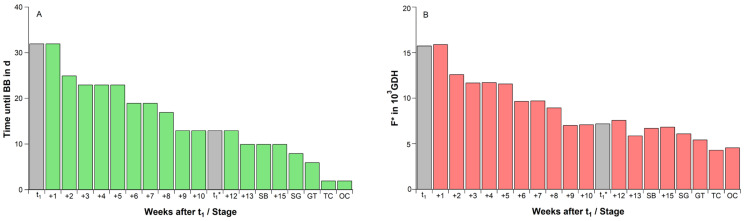
(**A**) Time until beginning of cherry blossom (BB) in days (d) and (**B**) forcing requirement between sampling and blossom (F* in GDH) for weekly sampling dates after t_1_ under controlled conditions (∼24 °C, 12 h light, 70% relative humidity), 2018/19 season. t_1_: endodormancy release, t_1_*: beginning of ontogenetic development. With beginning of bud development, sampling was done with development orientated at SB: ‘swollen bud’, SG: ‘side green’, GT: ‘green tip’, TC: ‘tight cluster’, OC: ‘open cluster’.

Twigs, which were sampled at t_1_, started to bloom (BBCH 60) in the climate chamber after 32 days (Figure 1A). The same was observed for the twigs, which were sampled one week later (340 DOY) and received 46 CP. However, from 347 DOY, the time until blossom gradually declined, finally to 13 days at t_1_* and 10 days at SB. Twigs collected after SB, had already started to develop in the orchard, so that now the time until flowering was reduced to less than 10 days. Under nearly constant forcing conditions, this led to a reduced forcing requirement until blossom (Figure 1B), which declined from 15,770 GDH at t_1_ to 7222 GDH at t_1_* (54% reduction) and to 6735 GDH at SB (58% reduction). 

When comparing the forcing requirement of buds until blossom from t_1_ and t_1_* in the climate chamber (F* = 15,770 resp. 7222 GDH, Figure 1B) with the 2018/19 forcing requirement in the orchard (F* = 4561 resp. 3850 GDH, Table 2), then a 3.5-fold higher forcing requirement until blossom from t_1_ and a 1.9-fold higher requirement from t_1_* in the climate chamber can be realized. However, it must be considered that the time until blossom in the climate chamber, under optimal forcing conditions (∼24 °C), was 32 days from t_1_ and only 13 days from t_1_*. The same periods lasted in the orchard 131 and 54 days, respectively (Table 1). This shows that the results derived from the climate chamber experiment cannot be directly transferred to the orchard, as the development and metabolism in the orchard is much slower. 

**Figure 2:**
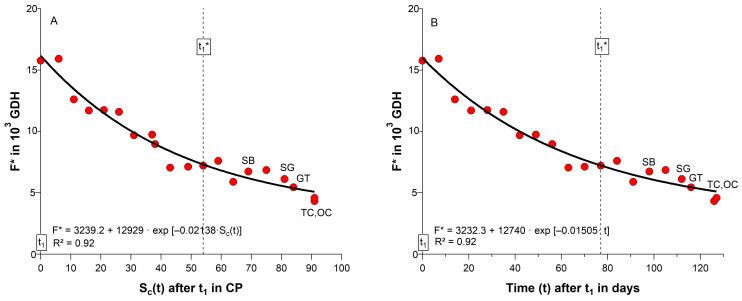
Relationship between forcing requirement until cherry blossom (F* in GDH) and (**A**) chill accumulation until sampling (S_c_(t) in CP), as well as (**B**) time after t_1_ in days, for cherry buds under controlled conditions (∼24 °C, 12 h light, 70% relative humidity) between t_1_ and ‘tight/open cluster’ (TC, OC), 2018/19 season. t_1_: endodormancy release, t_1_*: beginning of ontogenetic development, SB: ‘swollen bud’, SG: ‘side green’, GT: ‘green tip’, R^2^: coefficient of determination.

One common assumption is that additional chilling after endodormancy release reduces the forcing requirement until blossom. Table 2 already showed that during ecodormancy and ontogenetic development, temperatures in the orchard occur, which contribute to a further chill accumulation after t_1_. In the 2018/19 season, chill units raised from 40 CP at t_1_, to 94 CP at t_1_*, 109 CP at SB and 124 CP at GT. If we plot F* against the state of chilling S_c_(t) from t_1_ until OC, a significant negative exponential relationship between the weekly chill and forcing accumulation exist (R^2^ = 0.92, *p* ≤ 0.01; Figure 2A), which is frequently realized in parallel phenology models, where chill units are accumulated during ecodormancy or even during ontogenetic development, in order to calculate the forcing requirement until blossom (Equation (1)). According to this approach, a lack of chilling could be compensated by additional forcing and vice versa.

In the orchard data we did not find a reduction in F*(t_1_*–BB) (Table 2, Figure S2), whether related to the chill accumulation during ecodormancy (t_1_–t_1_*, r = 0.09^ns^) or for chill accumulation in the whole observation period (244–BB, r = −0.11^ns^). Figure S2 additionally indicates that in all 8 seasons forcing clearly reduces with the beginning of ontogenetic development (t_1_*), mainly after ‘swollen bud’, when the bud development has already started. Physiologically, the accumulation of chill units during ecodormancy is difficult to justify, since a cold stimulus is only assumed to release buds from endodormancy. Complicating is, that the timing of t_1_ is usually unknown. Figure 2B shows that the state of chilling can be easily replaced by the time after t_1_ (R^2^ = 0.92, *p* ≤ 0.01), indicating that the state of chilling during ecodormancy is not relevant for the subsequent forcing requirement F*, as already shown for the orchard data (Table 2).

In order to study if additional chilling during ecodormancy indeed reduces the time until cherry blossom for ‘Summit’, in 2018/19 a chilling/forcing experiment was carry out (Section 4.4). Since temperature in the climate chamber was nearly constant (∼24 °C), the time between sampling and blossom approximates the forcing requirement (F*).

**Figure 3:**
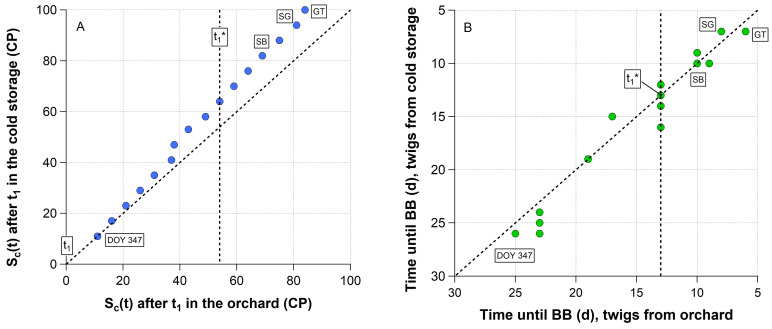
(**A**) Accumulated chill units after endodormancy release (t_1_) in chill portions (CP) in the orchard and cold storage between 347 DOY (13 December 2018, 14 days after t_1_) and 84 DOY (25 March 2019, 116 d after t_1_), (**B**) time until blossom at weekly intervals under constant forcing conditions (∼24 °C, 12 h light, 70% relative humidity), 2018/19 season. t_1_*: beginning of ontogenetic development, SB: ‘swollen bud’, SG: ‘side green’, GT: ‘green tip’.

In 2018/19, endodormancy was released on 29 November 2018 (333 DOY). At this time, buds in the orchard accumulated 40 CP (Table 2). In order to ensure that endodormancy is safely released, two weeks after t_1_ (347 DOY, buds now received 51 CP), 20 multi-branched twigs were cut from one ‘Summit’ tree. These twigs were placed in a cold storage, where they were exposed to a constant chilling temperature of t = 6.4 °C. As a result, the buds in the cold storage received at t_1_* 19% more ‘chill portions’ (64/54 CP) than the buds in the orchard (Figure 3A). However, up to this stage twigs taken weekly from the cold storage did not bloom significantly earlier under optimal forcing conditions than twigs taken from the orchard (Figure 3B). The difference in the blooming date ranged between both samples only between -2 and +3 days. With the beginning of ontogenetic development (45 DOY), it must be considered that the buds in the orchard and even in the cold storage slowly started to develop. On 7 March 2019 (66 DOY), the cherry trees in the orchard reached the stage ‘swollen bud’. However, only 3 days later the twigs in the cold storage were already in the stage ‘side green’. The stage ‘green tip’ was observed in the orchard on 25 March (84 DOY) and in the cold storage on 4 April (94 DOY). However, the low temperatures in the cold storage did not induce any bud development behind ‘green tip’, as long they were left there. In summary, the 19% additional chilling during ecodormancy in the cold storage did not clearly shorten the bud development until blossom under optimal forcing conditions.

### 2.4. Abscisic Acid Content in Cherry Flower Buds

Figure S3 shows the weekly abscisic acid (ABA) content of cherry flower buds for 8 seasons (2011/12–2018/19), from October of the previous year until ‘tight/open cluster’ of the flowering year. 

**Figure 4:**
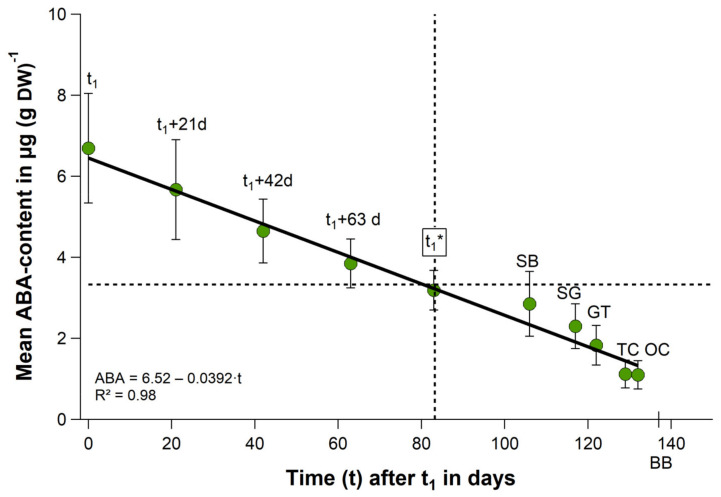
Mean ABA content of ‘Summit’ cherry flower buds from t_1_ until OC (2011/12–2018/19). Error bars show the standard deviation of 8 years, t_1_: endodormancy release, t_1_*: beginning of ontogenetic development, SB: ‘swollen bud’, SG: ‘side green’, GT: ‘green tip’ TC: ‘tight cluster’, OC: ‘open cluster’, BB: beginning of blossom, d: days, R^2^: coefficient of determination.

Each year from the beginning of October until ‘leaf fall’ in November, the ABA content in the buds raised, so that the maximum content was always observed during endodormancy phase (LF–t_1_). After endodormancy release in all seasons the value declined until ‘tight/open cluster’. On average there is nearly a linear reduction in the ABA content during this phase (Figure 4, Figure S3). The mean ABA content declined between t_1_ and t_1_* in 83 days (Table 1) from 6.69 ± 1.35 to 3.19 ± 0.49 μg/g DW at a rate of −0.042 μg/g DW per day by 52%. It further reduced from t_1_ until OC in 136 days to 1.10 ± 0.35 μg/g DW (83%). A similar reduction in the forcing requirement between t_1_ and t_1_* of 54% * was already stated in Section 2.3.

The annual ABA content at endodormancy release (t_1_) varied between 5.58 μg/g DW in 2014/15 and 9.04 μg/g DW in 2011/12 (Table 3). A hint for a time-dependent ABA reduction after endodormancy release (Figure 4) is its value at the beginning of ontogenetic development (t_1_*). The timing of t_1_* is very variable between the years as shown in Table 1 (CV = 30.6%). After a short duration of ecodormancy in the season 2014/15 (63 d), the ABA content reduced by only 40%, while it reduced after a long ecodormancy phase in 2012/13 (119 d) by 66% (r = 0.67, *p* = 0.071, Table 3).

**Figure 5:**
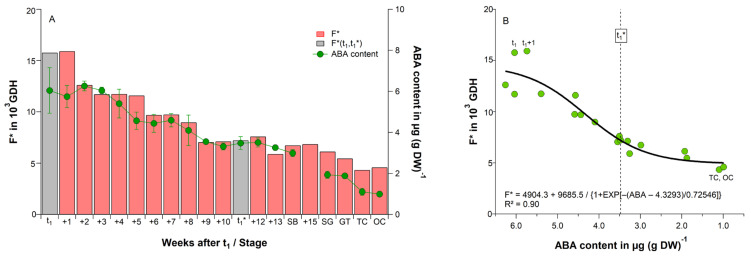
(**A**) Reduction in the forcing requirement under controlled conditions (∼24 °C, 12 h light, 70% relative humidity) until cherry blossom (F*) and ABA content of flower buds at sampling, between t_1_ and ‘tight/open cluster’ (TC, OC), 2018/19 season, r [F*, ABA] = 0.92, *p* ≤ 0.01), (**B**) relationship between F* and ABA content. t_1_: endodormancy release, t_1_*: beginning of ontogenetic development, R^2^: coefficient of determination.

The declining forcing requirement until cherry blossom in the 2018/19 forcing experiment (Section 2.3, Figure 1B) was well correlated with the bud’s ABA content at sampling (r = 0.92, *p* ≤ 0.01, Figure 5A). Figure 5B shows a sigmoid relationship between the forcing requirement of buds until blossom (F*) and the bud’s ABA content at sampling (R^2^ = 0.90, *p* ≤ 0.01). The decline of F* seems to be stronger for twigs, which were sampled during ecodormancy, compared to them taken during ontogenetic development (t_1_*–BB). This would support the relatively constant forcing requirement between t_1_* and BB (Table 2). Here, it must be considered that after t_1_*, the development of the buds has started and thus the heat requirement until flowering necessarily decreases stepwise.

In order to investigate what possibly reduces the ABA content in the cherry flower buds, firstly we investigated how additional chilling after t_1_ affected the ABA content. For this, the correlation between the weekly chill accumulation (ΔS_c_(t)) and changes in the ABA content (ΔABA, Figure S4) were calculated. Although chill units raised from week to week on average by 4.5 CP (range 0–14 CP), the weekly ABA content declined by 0.29 µg/g DW (range −1.42 until 1.18 µg/g DW). However, there was no significant correlation between both parameters in 8 years between t_1_ and OC, r (ΔABA, ΔS_c_(t)) = −0.15^ns^ (*n* = 152), as well as between t_1_ and t_1_* (r = −0.05^ns^, *n* = 93) and t_1_* and OC (r = −0.31^ns^, *n* = 59). 

**Figure 6:**
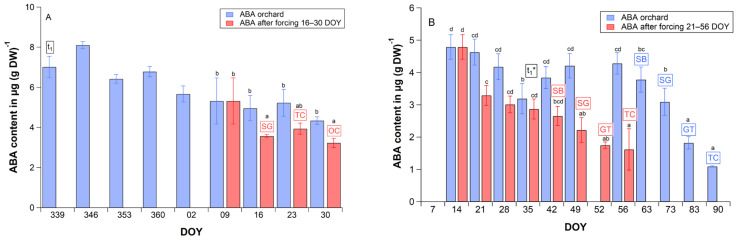
(**A**) Weekly ABA content of ‘Summit’ cherry flower buds (**A**) between endodormancy release (t_1_ = 339 DOY) and 30 DOY in the orchard (blue bars) and between 9 and 30 DOY under forcing conditions (red bars, ∼24 °C, 12 h light, 70% relative humidity) in 2019/20, (**B**) between 14 and 90 DOY in the orchard (blue bars) and between 14 and 56 DOY under forcing conditions (mean weekly temperature 8.0–12.0 °C, red bars) in 2013/14. t_1_*: beginning of ontogenetic development, SB: ‘swollen bud’, SG: ‘side green’, GT: ‘green tip’, TC: ‘tight cluster’, OC: ‘open cluster’. Error bars show the standard deviation of the repetitions (*n* = 3). Different letters indicate significant differences within the orchard and forcing experiment, ANOVA, Tukey-HSD test (*p* ≤ 0.05), DOY: day of year.

Secondly, we studied how additional forcing during ecodormancy influences the ABA content. For this, in the 2019/20 season the bud’s ABA content of trees in the orchard was compared with the ABA content of twigs under permanent forcing conditions (∼24 °C, 12 h light, 70% relative humidity) from 9 DOY until 30 DOY (Figure 6A, Section 4.5.). The mean ABA content of buds in the orchard declined after 9 January on average by 9% (until 16 DOY: 7%, 23 DOY: 2%, 30 DOY: 18%) and in the climate chamber significantly (*p* ≤ 0.01) by 33% (33%, 26%, 39%). This means, continued forcing reduced the ABA content by 33% in only 1 week (9–16 DOY) and enabled bud development, so that on 16 January 2020 the stage ‘side green’ was observed. This stage was detected in the orchard 8 weeks later (78 DOY). It must be emphasized that under forcing conditions the bud’s ABA content clearly reduced after 9 DOY without any further chill accumulation.

ABA data (not previously published) from a warming experiment in the 2013/14 season also support the results in Figure 6A. In this trial [31], three ‘Summit’ trees in the orchard were heated in transparent foil tents, bud development was observed and bud’s ABA content was analyzed for the heated and unheated trees. Moderate forcing temperatures in the foil tent (t = 10.3 °C, orchard: t = 2.0 °C) decreased the bud’s ABA content significantly (*p* ≤ 0.01) within 1 week (14–21 DOY) by 31%, compared to the orchard (Figure 6B). It further declined to 40% at ‘swollen bud’ (48.8% from t_1_) and to 66% at ‘tight cluster’ (71% from t_1_). This additionally confirms that the ABA content of buds reduced at warm environmental conditions. In the orchard, the bud’s ABA content started significantly (*p* ≤ 0.05) to decline 8 weeks later (63 DOY) at the ‘swollen bud’ stage.

## 3. Discussion 

### 3.1. Timing and Duration of Ecodormancy Phase

In all controlled phenological experiments, field studies and modelling approaches, it is important to know the timing of bud development, including the non-observable stages t_1_ and t_1_*. For the sweet cherry cv. Summit, these dates have been determined on the basis of climate chamber experiments (estimation of t_1_) and measurements of the bud’s water content (estimation of t_1_*) [8]. Thus, it was possible to derive the annual cycle of bud development for ‘Summit’ and its annual variability [10]. The timing of the non-observable ecodormancy phase was additionally confirmed by relevant metabolites, which showed significant changes in their course at the delimiting stages t_1_ and/or t_1_*. The ignorance of these relevant stages, which mark the ecodormancy phase, can lead to misinterpretations of experimental results and wrong conclusions. 

At this point, it has to be mentioned that after t_1_, buds need a long time before blossom (BBCH 60) can be observed (32 days in the 2018/19 forcing experiment, Figure 1A). The reason was the high ABA content in the buds, which is presented in this study for 8 seasons in weekly resolution. If the duration of the forcing experiments is too short (e.g., only 10–12 days), there is the risk that t_1_ and thus the endodormancy phase will be terminated too late or not recognized at all. For ‘Summit’, the phenological response after 13 days of optimal forcing (t~24 °C) indicated the beginning of ontogenetic development (Figure 1A). If they are not distinguishable between endo- and ecodormancy, this would be the end of winter rest. This shows that the individual assumptions in the forcing experiments are decisive for the achieved results. 

The low variability of the forcing requirement between t_1_* and BB (3447 ± 357 GDH, Table 2) indicated that t_1_* must be the right starting date for heat accumulation, which can be used in pure forcing models. In contrast, the accumulation of forcing units during ecodormancy was highly variable (CV = 74%, Table 2). A high or low amount of heat during this time did not result in an advanced or delayed blossoming date, so that the summation of GDH during ecodormancy is physiologically not recommended. This would also explain why the phenological model for cherry blossom, suggested by Chmielewski and Götz [31], performed relatively well at the experimental site in Berlin-Dahlem. The daylength term in the forcing model weighted the effectiveness of growing degree days between t_1_ and t_1_*. This is probably the reason, why Basler [32] in a model comparison of six tree-species concluded that the inclusion of photoperiod as driver after endodormancy release improved the leaf unfolding estimates and lead to more realistic model parameters. However, this study suggests that the daylength term in the model was only a substitution for the declining ABA content in the buds and the slowly rising effectiveness of forcing temperatures (GDD) toward t_1_*.

### 3.2. Chill and Forcing Compensation

In several controlled chilling/forcing experiments with seedlings, potted trees or cuttings, a substitution between chill and heat accumulation is supposed. Already Landsberg [19] and numerous further authors, cited in Cannel and Smith [17] and Wang et al. [23], described this inverse relationship, which is implemented in parallel phenology models. These experiments showed that after the exposure to continuous chill temperatures, the time until budburst or bloom and herewith the forcing requirement clearly reduced. Moreover, the forcing experiment in this study confirmed the reduction in the forcing requirement during ecodormancy from 15,770 GDH right after endodormancy release (t_1_) to 7222 GDH at the beginning of ontogenetic development (t_1_*), within 77 days (Figure 1B). Similar high forcing amounts between 13,000 and 11,000 GDH were found for three potted cherry cultivars, which have just been released from endodormancy by Kaufmann and Blanke [24]. Fadón et al. [26] stated that the cherry cv. Tamara, which received 23 CP in the field, required 22,390 GDH until full bloom (BBCH 65). With an increasing dwell time in the field, the heat required until blossom decreased, so that trees that received ~84 CP, before they were moved into a heated greenhouse, only needed 2845 GDH until blossom. It must also be considered that twigs were sampled after t_1_* already started to develop in the orchard, so that during ontogenetic development, F* must reduce (Figure 1B). Chill accumulation after t_1_* is continuous, but a physiological explanation of why it should be relevant during this phase is missing. Both studies concluded that additional heat can partially compensate low chill accumulation, an assumption that is also supported by Harrington et al. [20] and Pope et al. [28]. This conclusion would assume that a decreasing winter chill can be replaced by additional heat—a reassuring statement for fruit growers in the context of global warming. 

Menzel et al. [25], who also confirmed the negative exponential relationship between chilling and forcing in the framework of ‘citizen science’ experiments with *Corylus avellana* L. twigs stated that only the number of days, during which the branches were exposed to winter conditions, was sufficient to describe this compensatory effect. This means that chill accumulation can probably be replaced by time, which was also demonstrated in this study (Figure 2B). This result should stimulate further reflections on the physiological importance of chill accumulation during ecodormancy. In this study, it was shown that continuous chilling at 6.4 °C had no advancing effect on cherry blossom, compared to twigs that remained in the orchard and received 19% less chilling (Figure 3A). In both cases, the time until blossom reduced in a similar way, as shown in Figure 3B. Since we could still observe the ‘green tip’ stage in the cold storage, the temperature of 6.4 °C contributed to both chilling and, after a long stay in the cold storage, conditionally to moderate forcing. 

Compared to experiments under controlled conditions, only few field studies demonstrate the inverse relationship between winter chill and spring forcing [17,21,22]. In these studies, chill days (CD) were calculated from October/November and growing degree days (GDD) from January/February, both until budburst. These studies assume that chilling is relevant until budburst and forcing starts at a fixed date (1 January/February). Under these assumptions, a compensatory effect between chilling and forcing can be calculated. However, the dates of t_1_ and t_1_* were never considered in these statistical investigations. Wang et al. [23], who investigated the substitution between chilling and forcing for 30 perennials in Europe showed that positive chilling/forcing relationships can also be found, depending on the used chill model, which is a further uncertainty in these statistical studies. They also expressed doubts about whether this relationship is correctly interpreted in phenology models. Thus, Hänninen et al. [11] highlighted that the increasing trend to develop process-based phenology models, that are only based on phenological observations, run the risk of having no physiological background. Therefore, they called for more experimental studies to improve the models. 

### 3.3. ABA Content in Cherry Flower Buds

In none of the discussed experimental studies the bud’s ABA content was measured at sampling, so that this factor has not been considered to date. As already shown for five experimental years [8] and now in this study, the ABA content in sweet cherry flower buds increased from beginning of October to maximum values in November or the beginning of December, which can be assigned to the phase of endodormancy (LF–t_1_) (see also Table 3, Figure S3). This agrees with results in pear buds [13], where the ABA content peaked during endodormancy maintenance, rather than during the induction phase. The exposure to effective chilling temperatures takes place from beginning of September and continues during endodormancy, to fulfil the chilling requirement, for the cv. Summit on average of 42.6 CP (Table 2). Afterwards, when chilling requirement is fulfilled no further ABA is accumulated in the buds. From this point, ABA gradually decreases until the beginning of ontogenetic development (t_1_*) by ~50% (Table 3, Figure 4). Up to the stages ‘tight/open cluster’, the ABA content further reduces to ~80%, which is physiologically the result of the inactivation of ABA in the form of abscisic acid glucose ester (ABA-GE, Figure S5). Since ABA inhibits bud development, it can be assumed that heat accumulated shortly after t_1_ does not have the same effect on bud development as during ontogenetic development, when the bud’s ABA content is already reduced by about ~50%. This would generally explain the reduction in the forcing requirement or the declining dormancy depth during ecodormancy [38], which is, according to our findings (correlation analysis between ΔABA, ΔS_c_(t), Section 2.4.), not the result of additional chilling. On the contrary, this study has shown that even heat reduces the bud’s ABA content in absence of any chilling (Figure 6A). To our knowledge, this is the first study in which the ABA content of cherry buds has been systematically analyzed over 8 years. This gives us the certainty that the frequently stated chilling/forcing substitution during ecodormancy seems to be only an approximation for the declining bud’s ABA content in this phase. The relatively high ABA content after t_1_ prevents buds from too early a start of ontogenetic development, since these high forcing requirements, applied under controlled conditions, can never be reached in the orchard. Thus, the hypothesis of this study, that the declining ABA content in cherry flower buds during ecodormancy and not chilling reduces the heat requirement until blossom, can be confirmed and should result in an extension of ecodormancy definition. According to our findings, ecodormancy is a phase in which plant development is suppressed by low temperatures in the orchard and by a slowly declining ABA content in the flower buds. Thus, this phase is probably not only controlled by external factors, such as air temperature. 

### 3.4. Physiological Function of ABA

In autumn, the cold exposure increases the ABA content in buds by C-repeat binding factors (CBFs). These key-signaling genes can be rapidly induced by cold, can activate the transcription of DORMANCY-ASSOCIATED MADS-BOX (DAM) genes and a SHORT VEGETATIVE PHASE (SVP) homolog, can increase the ABA content and finally can establish endodormancy in buds [39]. As a result, the vessels of the vascular system in the buds are blocked by accumulated callose, a polysaccharide with the function of ‘temporary sealing material’ as a physical barrier [40], resulting in silenced cell division and cell expansion, metabolism and above all the blocked transport of growth promoting substances [39]. The significance of ABA in dormancy regulation is also evidenced by the precocious release of dormancy when ABA content in dormant buds is artificially reduced. It is widely accepted that ABA levels increase during dormancy establishment and decrease towards the transition from endodormancy to ecodormancy [34,37]. It is known that after endodormancy release, i.e., during ecodormancy, activating of the ABA catabolism takes place [39]. The reason is that homeostasis of ABA in plants is essential for normal growth and development, in which buds are both the target site for ABA to act upon and the principal location of ABA metabolism and catabolism [37]. Vimont et al. [34] reported for sweet cherry, that the expression for some genes involved in ABA biosynthesis steps (PavABA1a, PavABA1b, PavABA4b, PavNCED1, PavNCED4), were not correlated with ABA levels, whereas expression patterns of PavNCED3 and PavNCED9 genes shown a relationship to the ABA content. Using next generation sequencing and in-depth transcriptomic analyses showed complex arrays of signaling pathways for sweet cherry cultivars, including cold response genes, ABA, and oxidation-reduction processes [41]. 

As far as ABA catabolism is concerned [42], ABA-GE appears to be the major conjugate, which was found in various organs and cell organelles of different plant species [43]. The transport of ABA-GE is energy dependent and needs, therefore, adenosine triphosphate (ATP). The inactivation of ABA by glucose conjugation is reversible, and hydrolysis of ABA-GE catalyzed by ß-glucosidases results instantaneously in free ABA. The content of ABA-GE in the sweet cherry buds (Figure S5) was at the different stages and phases always markedly higher (range ~20–40 µg/g DW, ~15 µg/g DW at TC/OC), compared to the ABA content. This indicates that a great pool for the provision of ABA, but also as a storage pool in the buds exist. The ratio of ABA-GE/ABA was at LF and t_1_ similar with 4.6 and 5.3. However, during ecodormancy the decrease in the ABA content resulted in an increasing ratio of 8.5, which was doubled compared to LF and t_1_. At t_1_* this ratio achieved 12.6, and finally the maximum of 15.9 at TC/OC. These findings agree with results for sweet cherry varieties [34], where the content of ABA-GE was always higher compared with ABA.

ABA can also activate signaling factors (ABF2, ABF3, HB22), which in turn regulate the expression of DAM/SVP genes during endo- and ecodormancy ([39] and references therein). Since ABF2 is an essential component of glucose signaling [44], it can be assumed that ABA is influencing the energy providing processes, such as glycolysis and TCA cycle [10]. ‘Silencing’ takes place at high ABA contents, followed by a stepwise degradation, which result to an increasing expression of ABF2 and, therefore, released step-by-step these energy-yielding processes. For example, germination and seedling growth of Arabidopsis were significantly inhibited by ABF2 overexpression and inhibition was relieved by adding sucrose [44]. It should be also considered that ABA suppressed the expression of genes involved in photosynthesis, which starts when green tissues appear at the growth stage ‘side green’.

The conclusion from this study is that winter rest and bud development of perennial fruit trees cannot be described solely by air temperature (chilling, forcing). This finding is useful for predicting ecodormancy and subsequent heat accumulation until blossom. The study illustrates that physiological processes in the buds need to be better understood in order to avoid possible misinterpretations of the statistical or experimental results.

## 4. Material and Methods

### 4.1. Experimental Site

For this study, we used observational, experimental and analytic data from the sweet cherry orchard at Berlin-Dahlem (52.47° N, 13.30° E, h = 51 m) for 8 seasons (2011/12–2018/19). The orchard (980 m^2^) comprises 80 cherry trees (*Prunus avium* L.) of the cultivars ‘Summit’, ‘Regina’ and ‘Karina’, growing in 8 rows with 10 trees each, aligned in N–S direction. Trees are grafted on Gisela-5 rootstocks and pruning and irrigation was performed on demand. All investigations in this study are focused on ‘Summit’, of origin in British Columbia. The prevailing soil type is parabrown soil with weak marks of pale soil, FAO-Classification: Albic Luvisol. It is a silty to medium-loamy sand (surface soil) and silty-loamy sand to sandy clayey loam (sulesoil). The long-term annual mean air temperature and precipitation (1991–2020) are 10.4 °C and 562 mm, respectively.

### 4.2. Phenological Observation in the Orchard

In 8 seasons, the phenological stages ‘picking ripeness’ (PR: BBCH 87), ‘total leaf fall’ (LF: BBCH 97) and ‘beginning of blossom’ (BB: BBCH 60) were observed in the orchard, according to the BBCH scale [45,46]. Additionally, we registered the flower bud stages ‘swollen bud’ (SB: BBCH 51), ‘side green’ (SG: BBCH 53), ‘green tip’ (GT: BBCH 54), ‘tight cluster’ (TC: BBCH 55) and ‘open cluster’ (OC: BBCH 56). 

### 4.3. Determination of Ecodormancy Phase (t_1_–t_1_*)

The determination of the ecodormancy phase requires that the non-observable stages t_1_ and t_1_* are known. In order to estimate the beginning of ecodormancy, which starts with the date of endodormancy release (t_1_), we conducted climate chamber experiments (RUMED, Rubarth Apparate GmbH, Laatzen, Germany) for 8 seasons. Each season in November and December, 2 multi-branched ‘Summit’ twigs (~250 mm length, 5 mm diameter) with 2–3 bud clusters were cut weekly. After cutting, twigs were placed in 500 mL plastic flasks, filled with water to observe the beginning of blossom (BBCH 60) in a climate chamber with 12 h light, temperatures of ∼20/∼15 °C (day/night) and 70% relative humidity. Buds were observed daily to determine the beginning of blossom. When twigs successively reached the BBCH 60 stage under controlled conditions, we had the indication that the chill requirement in the orchard was fulfilled at sampling. Afterwards, we calculated the state of chilling S_c_(t) in chill portions (CP, [47,48,49]) from 1 September until the sampling date for the twigs, which started to bloom at first. For this, hourly temperatures were used, registered at the agrometeorological station in the vicinity of the orchard.

In order to determine the end of ecodormancy phase, which is marked by the beginning of ontogenetic development (t_1_*), the bud’s water content (WC) was analyzed weekly from 3 flower-bud cluster per tree, randomly over 4 trees (*n* = 4 replications), from beginning of October until April. In 8 seasons the water content, which was constant during ecodormancy (WC = 53.5%), started to rise continuously from a certain starting date until the stages ‘tight/open cluster’. This starting date was assigned to t_1_* because the rising water content was a clear sign for biological activity, which was related to continuously increasing temperatures [8].

### 4.4. Chilling and Forcing Demand under Controlled Conditions

In 2018/19, the climate chamber experiment (Section 4.3) was extended to investigate the compensatory effect of chill and forcing accumulation between t_1_ and OC at weekly intervals (Section 2.3, Figure 1 and Figure 2). With the onset of bud development in the orchard, twigs were collected developmentally oriented at SB, SG, GT, TC and OC. In order to force the bud development after endodormancy release effectively, the temperature in the climate chamber was set to ∼24 °C, 12 h light and 70% relative humidity. Hourly temperatures and humidity in the climate chamber were recorded. The timing of the BBCH 60 stage was registered, in order to calculate the time between sampling and blossom in days and the forcing requirement until blossom in growing degree hours (GDH, [50]). We also considered the forcing amount that the plant already accumulated in the orchard. Chill units, that the twigs received until sampling in the orchard, were calculated in chill portions.

Additionally, in the 2018/19 season we cut on 347 DOY (two weeks after endodormancy release) 20 twigs with several flower bud clusters and stored them in a water bucket at a constant temperature of t = 6.4 °C. These twigs, which received continuously chilling temperatures, were also weekly transferred to the climate chamber, alongside the twigs from the orchard. This experiment allowed us to study the effect of additional chilling on cherry bud development until blossom (Section 2.3, Figure 3).

### 4.5. Targeted Analysis of the Abscisic Acid (ABA) Content in Flower Buds

In order to analyze the ABA content in the flower buds, in each season between LF and OC, three bud clusters of four (2011/12–2017/18) and three trees each (2018/19) were taken weekly in the orchard at random locations over the whole tree. After the beginning of bud development sampling was done at the stages SB, SG, GT, TC and OC. After cutting, clusters were immediately placed in plastic bags on ice in a polystyrene box. They were consequently frozen in liquid nitrogen and stored at −80 °C until freeze-drying. All buds were ground in a ball mill (Retsch M1, Haan, Germany) before analysis. The ABA content was analyzed in a targeted assay by Metabolon Inc., 617 Davis Drive, Morrisville, NC 27560 (www.metabolon.com, accessed on 1 August 2022), along with further relevant metabolites, including ABA-glucosyl ester [10].

In the 2019/20 season, the bud’s ABA content of twigs after 1 to 3 weeks of pure forcing between 9 and 30 DOY (climate chamber, ∼24 °C, 12 h light and 70% relative humidity) was analyzed as described above and compared with the ABA content of buds at the same time in the orchard (Section 2.4, Figure 6A).

### 4.6. Statistical Analysis

Standard statistical analyses, including the calculation of mean values, standard deviations, Pearson correlation coefficient and linear/non-linear regression functions, were performed in IBM SPSS Statistics V25. Significance of temporal changes of the ABA and ABA-GE content was tested with the Tukey HSD test (Figure 6A,B and Figure S5, *p* ≤ 0.05). Figures and some statistical calculations were performed with IGOR Pro V6.3.7.2.

## Figures and Tables

**Table 1:** Timing of ‘total leaf fall’ (LF), endodormancy release (t_1_), beginning of ontogenetic development (t_1_*), beginning of blossom (BB) and duration of endo- (LF–t_1_), ecodormancy phase (t_1_–t_1_*) and ontogenetic development (t_1_*–BB) for ‘Summit’, 2011/12–2018/19. x: mean, s: standard deviation, CV: coefficient of variation (%), DOY: day of year.

	LF in DOY	t_1_ in DOY	t_1_* in DOY	BB in DOY	Duration (LF–t_1_) in d	Duration (t_1_–t_1_*) in d	Duration (t_1_*–BB) in d
2011/12	312	335	45	105	23	75	60
2012/13	304	332	85	116	28	119	31
2013/14	302	323	35	95	21	77	60
2014/15	322	343	41	111	21	63	70
2015/16	307	328	61	111	21	98	50
2016/17	313	341	45	97	28	70	52
2017/18	305	340	59	108	35	84	49
2018/19	312	333	45	99	21	77	54
Date	11/06	11/30	02/21	04/15	-	-	-
**x**	**309.6**	**334.4**	**52.0**	**105.3**	**24.8**	**82.9**	**53.3**
s	6.5	6.8	15.9	7.6	5.1	17.8	11.3
CV	2.1	2.0	30.6	7.2	20.8	21.5	21.2

Correlation: r [t_1_*, BB] = 0.76, *p* ≤ 0.05.

**Table 2:** Chill (CA) and forcing (FA) accumulation in different phases, 2011/12–2018/19; 1 September—endodormancy release (DOY 244–t_1_), ecodormancy phase (t_1_–t_1_*), ontogenetic development (t_1_*–BB). t_1_: endodormancy release, t_1_*: beginning of ontogenetic development, BB: beginning of blossom x: mean, s: standard deviation, CV: coefficient of variation (%), CP: chill portions, GDH: growing degree hours.

Season	CA (244–t_1_) in CP	CA (t_1_–t_1_*) in CP	CA (t_1_*–BB) in CP	CA (244–BB) in CP	FA (t_1_–t_1_*) in GDH	FA (t_1_*–BB) in GDH	FA (t_1_–BB) in GDH
2011/12	42	40	50	132	642	3029	3671
2012/13	43	57	17	117	645	3315	3960
2013/14	40	51	42	133	802	3664	4466
2014/15	40	41	49	130	744	3290	4034
2015/16	41	67	36	144	2460	3050	5510
2016/17	46	37	39	122	312	3378	3690
2017/18	49	53	29	131	763	3998	4761
2018/19	40	54	38	132	711	3850	4561
**x**	**42.6**	**50.0**	**37.5**	**130.1**	**884.9**	**3446.8**	**4331.6**
s	3.3	10.1	10.7	8.0	654.4	357.0	623.2
CV	7.7	20.2	28.6	6.1	74.0	10.4	14.4

Correlations: r [CA (t_1_–t_1_*), FA (t_1_–t_1_*)] = 0.76, *p* ≤ 0.05; r [CA (t_1_–t_1_*), FA (t_1_*–BB)] = 0.09^ns^; r [CA (244–BB), FA (t_1_*–BB)] = −0.11^ns^.

**Table 3:** ABA content at phenological stages, duration of ecodormancy (t_1_–t_1_*) and ABA reduction (%) from t_1_ until t_1_* and OC, 2011/12–2018/19. x: mean, s: standard deviation, t_1_: endodormancy release, t_1_*: beginning of ontogenetic development, OC: ‘open cluster’.

Season	ABA(t_1_) μg/g DW	ABA(t_1_*) μg/g DW	ABA(OC) μg/g DW	Duration (t_1_–t_1_*) in d	ABA Reduction in %	
					(t_1_–t_1_*)	(t_1_–OC)
2011/12	9.04	3.19	0.93	75	64.7	89.7
2012/13	6.04	2.07	0.85	119	65.8	86.0
2013/14	5.60	3.19	1.09	77	43.0	80.5
2014/15	5.58	3.37	1.22	63	39.6	78.1
2015/16	8.60	3.40	0.89	98	60.5	89.7
2016/17	6.19	3.15	0.92	70	49.1	85.2
2017/18	6.46	3.71	1.90	84	42.7	70.6
2018/19	6.04	3.48	0.99	77	42.3	83.5
**x**	**6.69**	**3.19**	**1.10**	**82.9**	**51.0**	**82.9**
s	1.35	0.49	0.35	17.8	10.9	6.4

Correlation: r [duration (t_1_–t_1_*), ABA reduction (t_1_–t_1_*)] = 0.67, *p* = 0.071.

## Data Availability

Not published data, related to this paper, are available on reasonable request to the corresponding authors.

## Supplementary Materials

The following supporting information can be downloaded at: https://www.mdpi.com/article/10.3390/plants11152044/s1, Figure S1: Chill accumulation S_c_(t) in chill portion (CP) between 1 September and endodormancy release (t_1_) as well as hourly forcing units S_F_(t) in growing degree hours (GDH) during ecodormancy (t_1_–t_1_*) and ontogenetic development (t_1_*-BB) in the experimental sweet cherry orchard Berlin-Dahlem, seasons 2011/12–2018/19. t_1_*: beginning of ontogenetic development, BB: Beginning of blossom (BBCH 60); Figure S2: State of chilling S_c_(t) in chill portions (CP) from 1 September until ‘open cluster’ (blue line) and heat requirement F* until cherry blossom (red line) in the experimental sweet cherry orchard Berlin-Dahlem, seasons 2011/12–2018/19. Presented are weekly data, after ‘swollen bud’ (SB) development orientated for ‘side green’ (SG), ‘green tip’ (GT), ‘tight cluster’ (TC), ‘open cluster’ (OC). LF: ‘total leaf fall’, t_1_: endodormancy release, t_1_*: beginning of ontogenetic development. The horizontal dashed line indicates the forcing requirement until blossom from t_1_*; Figure S3: ABA content of sweet cherry flower buds (cv. Summit) between beginning of October (previous year) and ‘open cluster’ of the flowering year in the season 2011/12–2018/19. LF: ‘total leaf fall’, t_1_: endodormancy release, t_1_*: beginning of ontogenetic development, Stages after ‘swollen bud’ (SB): ‘side green’, ‘green tip’, ‘tight cluster’, ‘open cluster’ (OC); Figure S4: ABA content of sweet cherry flower buds (cv. Summit) between beginning of October (previous year) and ‘open cluster’ of the flowering year and chill accumulation S_c_(t) in chill portions (CP) between endodormancy release (t_1_) and ‘open cluster’ in the season 2011/12–2018/19. LF: total leaf fall, t1: endodormancy release, t_1_*: beginning of ontogenetic development, Stages after ‘swollen bud’ (SB): ‘side green’, ‘green tip’, ‘tight cluster’, ‘open cluster’ (OC); Figure S5: ABA and ABA-GE content of sweet cherry buds (cv. Summit) at selected phenological stages and during ecodormancy, in the season 2011/12–2018/19. LF: ‘total leaf fall’, t_1_: endodormancy release, t_1_*: beginning of ontogenetic development, ‘tight cluster’ (TC), ‘open cluster’ (OC), ecodormancy phase (t_1_–t_1_*). Different letters indicate significant differences, ANOVA, Tukey-HSD test (*p* ≤ 0.05).
