# Supplementary material for: ABA and Not Chilling Reduces Heat Requirement to Force Cherry Blossom after Endodormancy Release
Source: Plants (Basel). 2022 Aug 4;11(15):2044. doi: 10.3390/plants11152044 (PMC9370221; doi:10.3390/plants11152044)
Supplement: Supplementary file 1 [file plants-11-02044-s001.zip › plants-1831909-supplementary.pdf]

Supplementary Figures

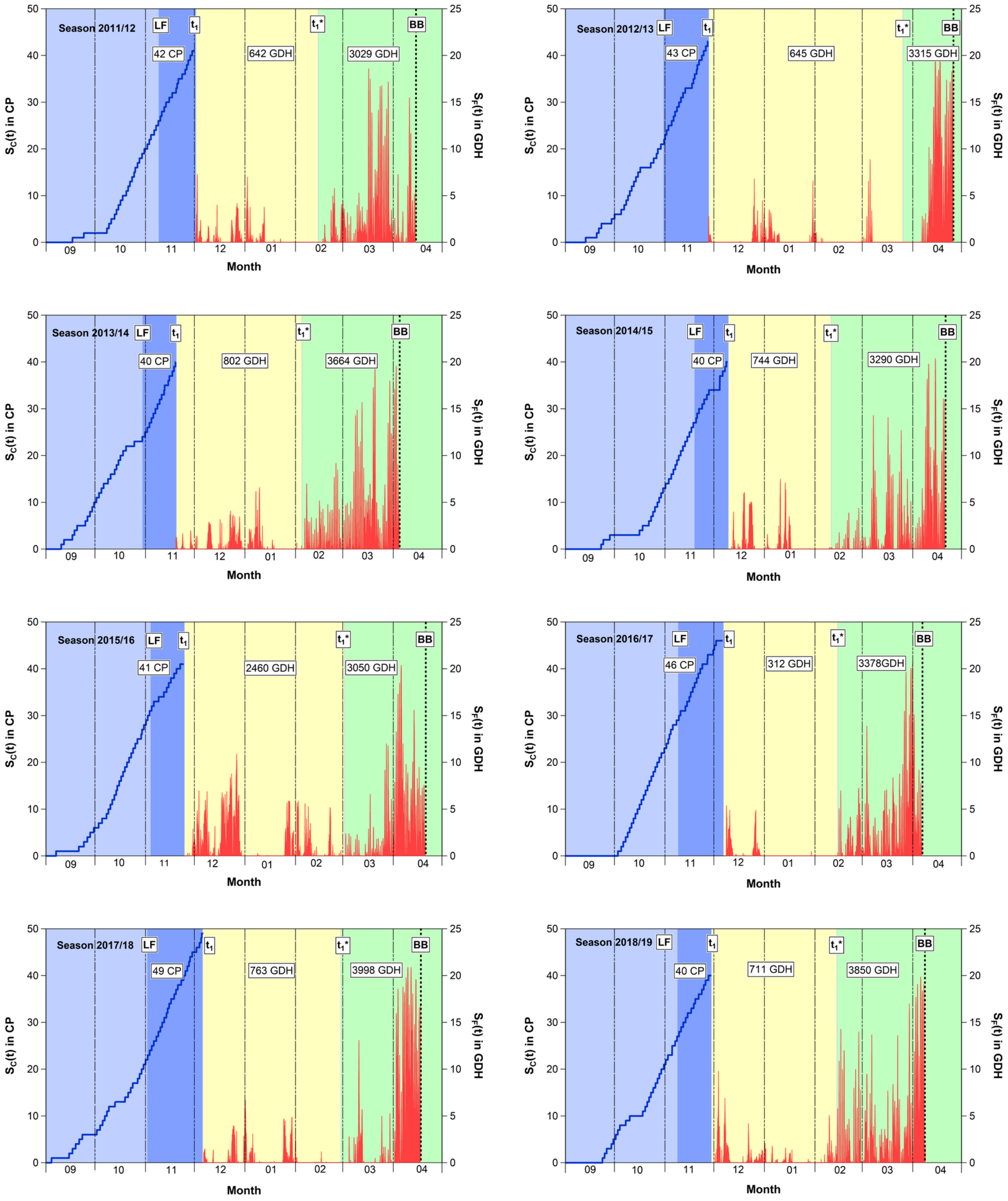

Figure S1. Chill accumulation $S_c(t)$ in chill portion (CP) between 1 September and endodormancy release (t_1) as well as hourly forcing units $S_F(t)$ in growing degree hours (GDH) during ecodormancy (t_1-t_1*) and ontogenetic development (t_1*-BB) in the experimental sweet cherry orchard Berlin-Dahlem, seasons 2011/12-2018/19. t_1*: beginning of ontogenetic development, BB: Beginning of blossom (BBCH 60).

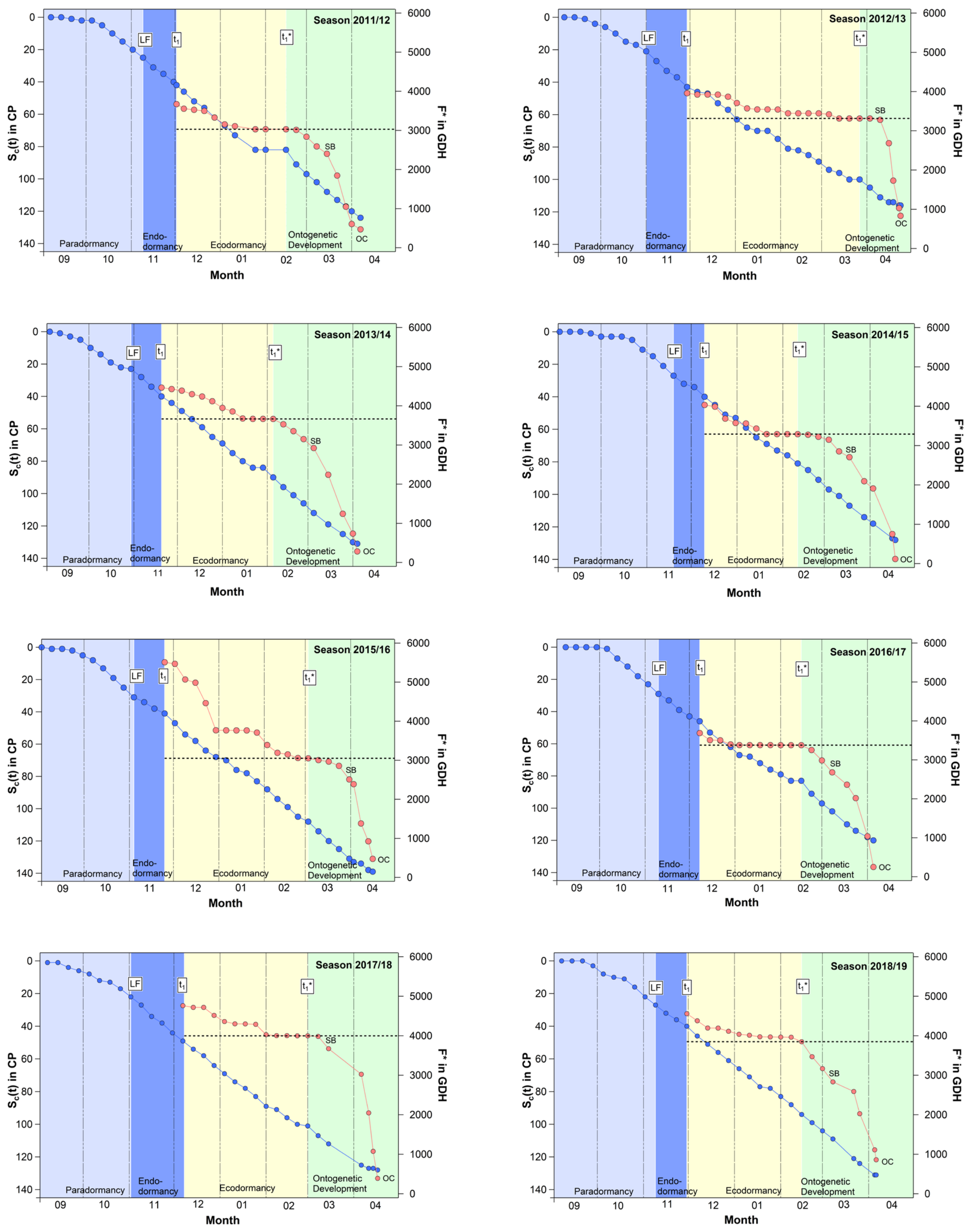

Figure S2. State of chilling $S_c(t)$ in chill portions (CP) from 1 September until 'open cluster' (blue line) and heat requirement F* until cherry blossom (red line) in the experimental sweet cherry orchard Berlin-Dahlem, seasons 2011/12-2018/19. Presented are weekly data, after 'swollen bud' (SB) development orientated for 'side green' (SG), 'green tip' (GT), 'tight cluster* (TC), 'open cluster' (OC). LF: 'total leaf fall', t_1: endodormancy release, t_1*: beginning of ontogenetic development. The horizontal dashed line indicates the forcing requirement until blossom from t_1*.

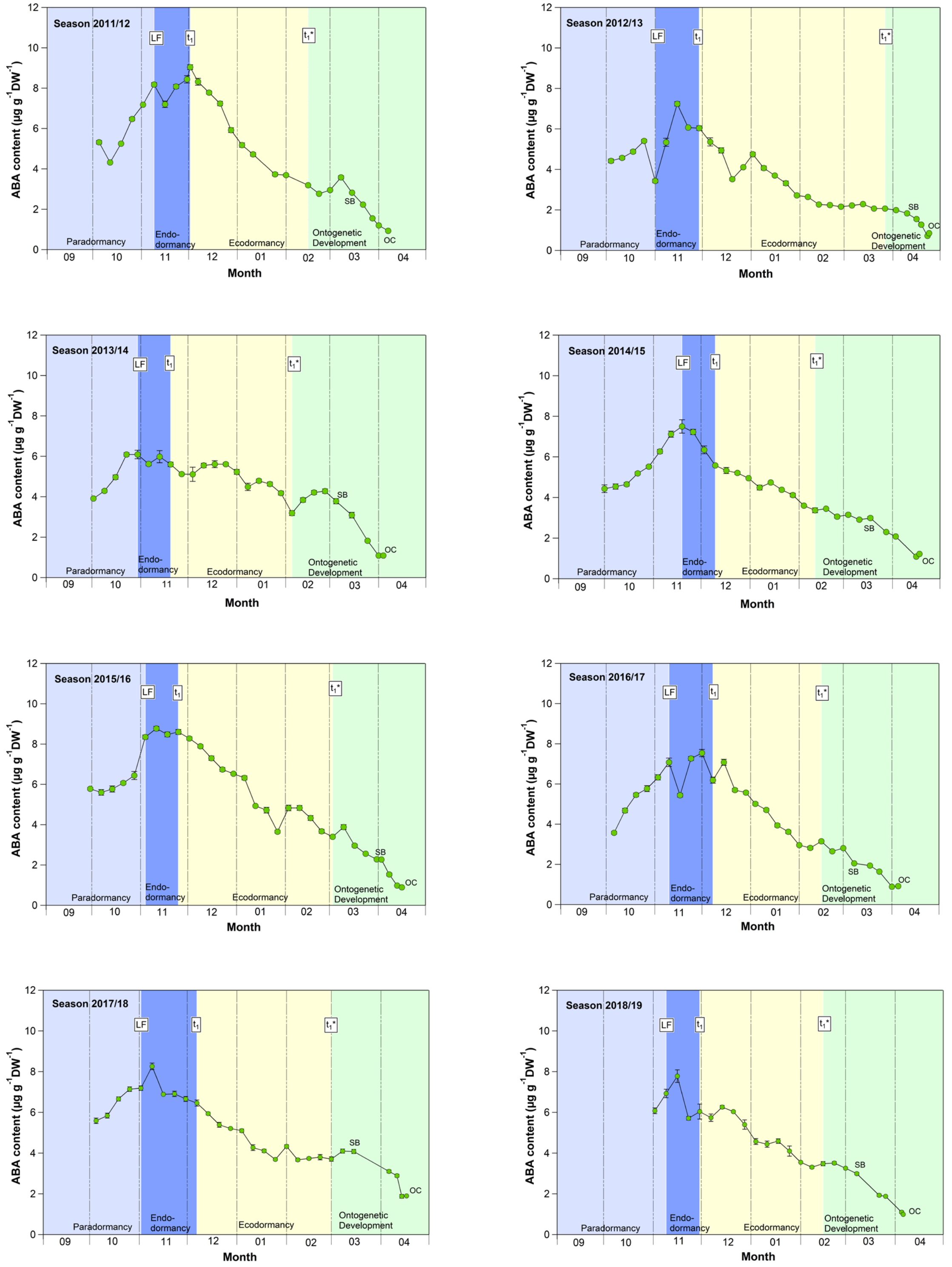

Figure S3. ABA content of sweet cherry flower buds (cv. Summit) between beginning of October (previous year) and 'open cluster' of the flowering year in the seasons 2011/12-2018/19. LF: 'total leaf fall', t_1: endodormancy release, t_1*: beginning of ontogenetic development, Stages after 'swollen bud' (SB): 'side green', 'green tip', 'tight cluster', 'open cluster' (OC).

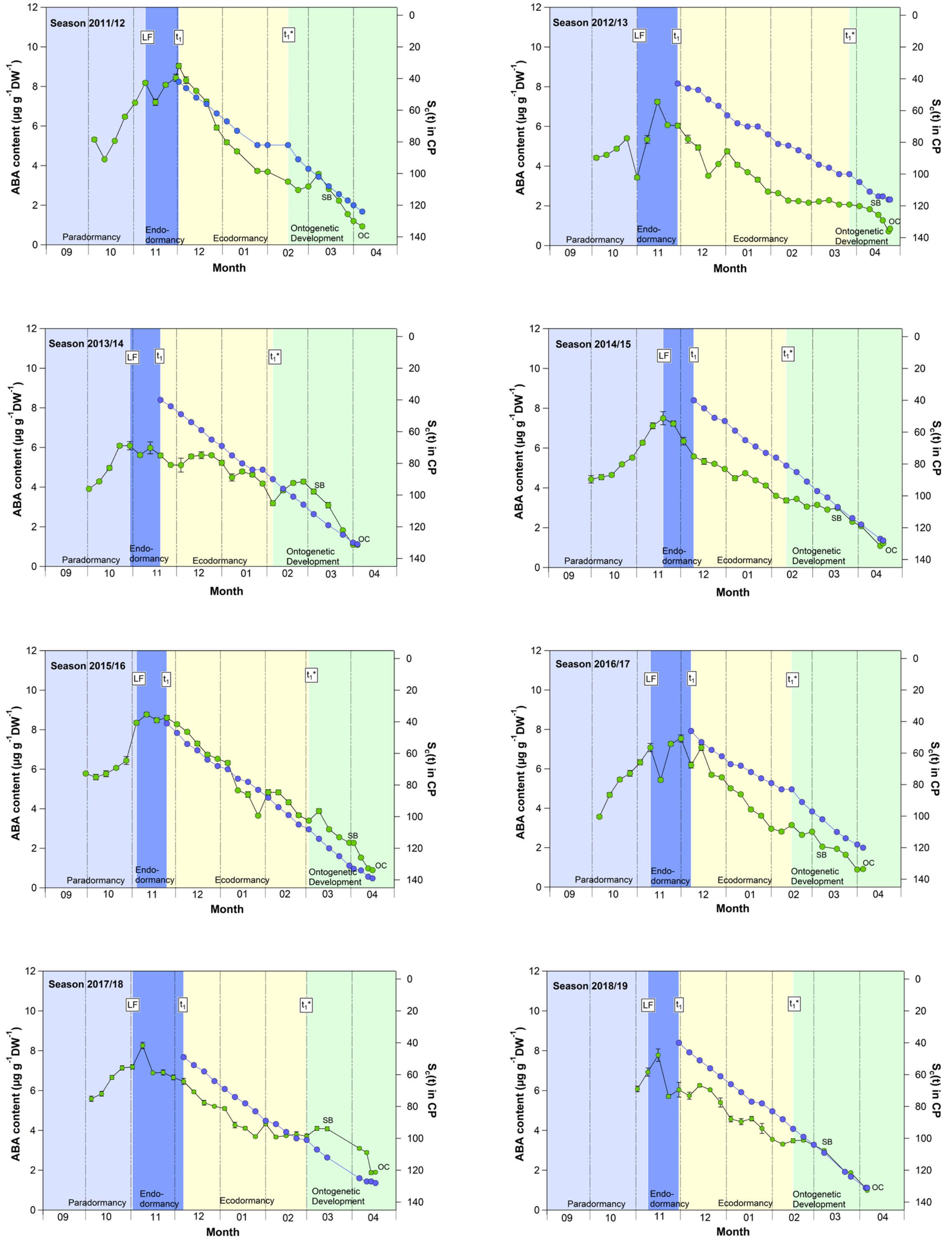

Figure S4. ABA content of sweet cherry flower buds (cv. Summit) between beginning of October (previous year) and 'open cluster' of the flowering year and chill accumulation $S_c(t)$ in chill portions (CP) between endodormancy release (t_1) and 'open cluster' in the season 2011/12-2018/19. LF: 'total leaf fall', t_1: endodormancy release, t_1*: beginning of ontogenetic development, Stages after 'swollen bud' (SB): 'side green', 'green tip', 'tight cluster', 'open cluster' (OC).

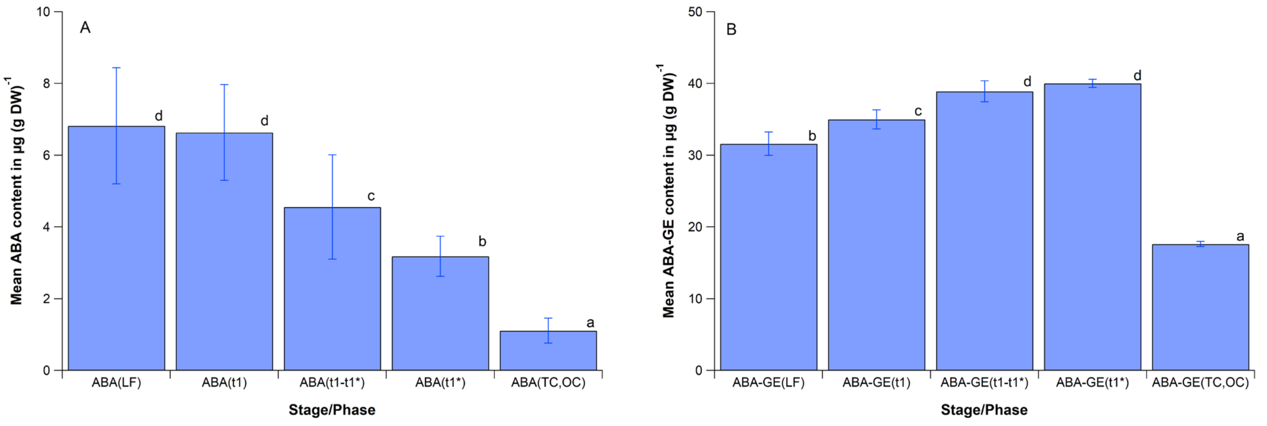

Figure S5. ABA and ABA-GE content of sweet cherry buds (cv. Summit) at selected phenological stages and during ecodormancy, in the season 2011/12-2018/19. LF: 'total leaf fall', t_1: endodormancy release, t_1*: beginning of ontogenetic development, 'tight cluster' (TC), 'open cluster' (OC), ecodormancy phase (t_1-t_1*).
